# Screening of *Satureja subspicata* Vis. Honey by HPLC-DAD, GC-FID/MS and UV/VIS: Prephenate Derivatives as Biomarkers

**DOI:** 10.3390/molecules21030377

**Published:** 2016-03-21

**Authors:** Igor Jerković, Marina Kranjac, Zvonimir Marijanović, Marina Zekić, Ani Radonić, Carlo Ignazio Giovanni Tuberoso

**Affiliations:** 1 Department of Organic Chemistry, Faculty of Chemistry and Technology, University of Split, Ruđera Boškovića 35, Split 21000, Croatia; mkranjac@ktf-split.hr (M.K.); zekic@ktf-split.hr (M.Z.); radonic@ktf-split.hr (A.R.); 2 Department of Food Technology, Marko Marulić Polytechnic in Knin, Petra Krešimira IV 30, Knin 22300, Croatia; zmarijanovic@veleknin.hr; 3 Department of Life and Environmental Sciences, University of Cagliari, via Ospedale 72, Cagliari 09124, Italy; tuberoso@unica.it

**Keywords:** *Satureja subspicata* Vis. honey, GC-FID/MS, HPLC-DAD, UV/Vis spectrometry, prephenate derivatives, methyl syringate

## Abstract

The samples of *Satureja subspicata* Vis. honey were confirmed to be unifloral by melissopalynological analysis with the characteristic pollen share from 36% to 71%. Bioprospecting of the samples was performed by HPLC-DAD, GC-FID/MS, and UV/VIS. Prephenate derivatives were shown to be dominant by the HPLC-DAD analysis, particularly phenylalanine (167.8 mg/kg) and methyl syringate (MSYR, 114.1 mg/kg), followed by tyrosine and benzoic acid. Higher amounts of MSYR (3–4 times) can be pointed out for distinguishing *S. subspicata* Vis. honey from other *Satureja* spp. honey types. GC-FID/MS analysis of ultrasonic solvent extracts of the samples revealed MSYR (46.68%, solvent pentane/Et_2_O 1:2 (*v*/*v*); 52.98%, solvent CH_2_Cl_2_) and minor abundance of other volatile prephenate derivatives, as well as higher aliphatic compounds characteristic of the comb environment. Two combined extracts (according to the solvents) of all samples were evaluated for their antioxidant properties by FRAP and DPPH assay; the combined extracts demonstrated higher activity (at lower concentrations) in comparison with the average honey sample. UV/VIS analysis of the samples was applied for determination of CIE Lab colour coordinates, total phenolics (425.38 mg GAE/kg), and antioxidant properties (4.26 mmol Fe^2+^/kg (FRAP assay) and 0.8 mmol TEAC/kg (DDPH assay)).

## 1. Introduction

It has been pointed out that targeted analysis of honey’s natural organic compounds could be a useful tool for characterization of its botanical origin accompanying melissoplaynological analysis and other routine physical-chemical parameters [1,2,3]. A large biodiversity of natural organic compounds derived from different biosynthetic pathways has been found in honey [1]: carbohydrates, nitrogen containing compounds, phenolics, volatiles (e.g., monoterpenes, norisoprenoids or benzene derivatives), and others. The honey compounds have been found to be the plant-derived constituents (phytochemicals) in their native form or transformed by the bees, the compounds generated by the bees or transferred from the combs, but also the products of thermal treatment and/or prolonged storage (derived from Maillard reactions and Strecker degradations) as well as microbial or environmental contaminants. Our recent research has been focused on the bioprospecting of different honey types in order to characterize the samples by screening the organic compounds to find characteristic chemical profiles, chemical biomarkers of the honey botanical origin, or bioactive compounds [4,5,6].

The genus *Satureja* is comprised of *ca.* 200 species, and in Croatian flora, four species are distributed in Mediterranean region: *S. montana* L., *S*. *cuneifolia* Ten., *S.* *subspicata* Vis., and an endemic *S. visianii* Šilić [7,8,9]. Due to the presence of secondary metabolites such as flavonoids, steroids, essential oils, or tannins, *Satureja* species have been known for their healing properties and have been used as traditional folk remedies [9] with antimicrobial activity, anti-inflammatory, and anti-nociceptive effects [10,11]. Rutin, quercetin, caffeic, *p*-coumaric, ellagic, protocatechuic, rosmarinic, and syringic acids were identified and quantified in *S.* *subspicata* Vis. of Croatian origin using HPLC-DAD [12]. The results showed that its extracts contain polyphenolics and other antioxidant compounds with chelating and radical scavenging properties. Carvacrol, α-pinene, *p*-cymene, γ-terpinene, and thymol methyl ether appeared as the major components of the essential oil of *S. subspicata* Vis., followed by smaller percentages of myrcene, linalool, β-caryophyllene, limonene, geranyl acetate, *oct*-1-en-3-ol, nerol, thymol, and borneol [9]. *Satureja* species are also nectariferous plants. Our previous research has been focused on *S. montana* L. and *S. hortensis* L. honey types [13,14]. However, *S. subspicata* Vis. honey has not yet been investigated. Therefore, the objectives of the present bioprospecting study (which is a first time report, to our best knowledge) were: (i) targeted HPLC-DAD analyses of native unifloral *S. subspicata* Vis. honey samples focused on characteristic non-volatiles; (ii) GC-FID/MS analyses of the ultrasonic solvent extracts of the samples directed toward volatile/semi-volatile organic compounds and evaluation of their 2,2-diphenyl-1-picrylhydrazyl (DPPH) radical scavenging ability and ferric reduction activity potential (FRAP); (iii) U*V*/*V*IS determination of colour, total phenolic content, and antioxidant properties (DPPH and FRAP assays) of the samples; (iv) the comparison of the obtained results with our other reports on different *Satureja* spp. honey types or other honeys of different botanical origin.

## 2. Results and Discussion

All the samples of *S. subspicata* Vis. honey were unifloral according to the melissopalynological analysis, with *Satureja subspicata* Vis. pollen share in the range from 36% to 71%. Accompanying plant species were also determined: *Centaurea jacea* L. (2%–19%), *Rhamnus* spp. (0%–19%), Apiaceae (0%–13%), Fabaceae (0%–7%), *Melilotus* spp. (0%–6%), *Trifolium* spp. (0%–4%), Asteraceae (0%–3%), *Centaurea cyanus* L. (0%–2%), *Eryngium* spp. (0%–2%), Brassicaceae (0%–2%), *Scabiosa* spp. (0%–2%), and Liliaceae (0%–2%). The samples were directly analysed by HPLC-DAD and UV/VIS, while the obtained ultrasonic solvent extracts were analysed by GC-FID/MS and two combined extracts (according to the solvents) of all samples by UV/VIS (for research of their antioxidant properties).

### 2.1. HPLC-DAD Analysis of Native Honey Samples

The compounds identified in *Satureja subspicata* Vis. native honey samples by targeted HPLC analysis are presented in Table 1. Prephenate derivatives dominated (particularly phenylalanine, methyl syringate, tyrosine, and benzoic acid). Methyl syringate (MSYR) was found at high amounts (114.1 ± 17.8 mg/kg). It was highlighted in our previous papers [13,14] as non-specific chemical marker of different *Satureja* honeys (27.98 ± 12.95 mg/kg of *Satureja montana* L. honey samples and 39.32 mg/kg of *Satureja hortensis* L. honey sample). However, the 3–4 times higher amount of MSYR found in this present research can be pointed out for distinguishing *S. subspicata* Vis. honey from other *Satureja* spp. honeys. A twofold higher quantity of MSYR (229.2 ± 57.9 mg/kg) was found in Asphodel monofloral honey [15] and indicated that the chemical marker originated from the nectar (with an average contribution of the nectar to the honey of 80%). MSYR was also detected in manuka honey [16] at higher levels (45 mg/kg). Lower MSYR levels (0.093 to 5.044 mg/kg) were found and dosed in the honeys and honeydews derived from other botanical sources, such as willow [17], cornflower [18], robinia, rape, chestnut, clover, linden blossom, dandelion, sunflower, and fir [19].

Besides leptosin, MSYR was identified as the active compound from manuka honey to which its myeloperoxidase (MPO)-activity inhibition is ascribed [20]. MSYR displays the scavenging activity of superoxides and inhibitory effects on aflatoxin production [21,22]. It is also a selective agonist [23,24] of transient receptor potential channel ankryn 1 (TRPA1), and an efficient phenolic mediator for bacterial and fungal laccases [25].

Two aromatic acids, benzoic acid (36.4 ± 10.5 mg/kg) and 4-hydroyphenylacetic acid (21.3 ± 8.1 mg/kg), as well as kojic acid (5-hydroxy-2-(hydroxymethyl)-4*H*-pyran-4-one; 13.7 ± 1.2 mg/kg), were also identified in the samples, but in lower concentrations. Partial qualitative similarity with manuka honey is again noticed. Namely, three chemotypes of *Leptospermum scoparium* honey were identified [20] containing: (i) high levels of 4-hydroxybenzoic acid, dehydrovomifoliol, and benzoic acid; (ii) high concentrations of kojic acid and 2-methoxybenzoic acid and (iii) high contents of syringic acid, 4-methoxyphenyllactic acid, and MSYR. Kojic acid was also detected and dosed [4] by HPLC-DAD in *Coffea* spp. honey. 4-Hydroxyphenylacetic acid is involved in the scavenging reactive oxygen and nitrogen species, both *in vitro* and *in vivo,* with lower potential in comparison with other more hydroxylated aromatic acids [26,27]. Its antioxidant properties were expressed as the ability to scavenge the free radical ABTS·+ (IC_50_(μM) = 852.713) and as TEAC (Trolox equivalent antioxidant capacity, 0.030 (μM)). Kojic acid inhibits catecholase activity of tyrosinase [28]. It is a weak bacterial antagonist, its range of activity being in a dilution of 1:1.000 to 2.000 against the common test bacteria. The MIC (minimum inhibitory concentration) of kojic acid against the human pathogenic fungi was in the range 80–640 µg/mL.

Other detected and dosed aromatic acids in the samples were amino acids phenylalanine (167.8 ± 80.8 mg/kg) and tyrosine (35.6 ± 14.8 mg/kg). The amounts of these amino acids found in one sample of *S. hortensis* L. were [14]: phenylalanine 282.0 mg/kg and tyrosine 140.4 mg/kg; there is no data of their distribution in *S. montana* L. honey. The tyrosine amount was similar to eucalyptus honey (30.48 mg/kg) and orange blossom honey (31.5 mg/kg), while phenylalanine content was close to eucalyptus honey (107 mg/kg) and orange blossom honey (150 mg/kg), as previously reported [29]. In general, proteins and amino acids in the honey are attributable to animal and vegetal sources, the major of these being pollen [29]. The major amino acids found in rosemary, eucalyptus, lavender, thyme, and orange blossom honeys were proline (the major contributor), phenylalanine, tyrosine, and lysine.

### 2.2. GC-FID/MS Analysis of the Ultrasonic Solvent Extracts

Two solvents were used for the ultrasonic solvent extraction of *S. subspicata* Vis. honey samples in order to obtain more detailed chemical profiles of the volatile/semi-volatile compounds. The solvents differ in polarity, and consequently could influence the extraction of the compounds according to their polarity and water-solubility. The extracts were analysed by GC-FID/MS, and the results are presented in Table 2. However, the extracts, regardless of the solvent used, were qualitatively very similar, with smaller qualitative differences among minor compounds. The major identified compound was MSYR (46.68%; 52.98%), followed by syringaldehyde (7.30%; 8.80%), and benzoic acid (2.22%; 1.76%). Other structurally related compounds were found with minor ranges (Table 2) such as: 3,4,5-trimethylphenol, eugenol, methyl 4-methoxybenzoate, 3,4,5-trimethoxybenzaldehyde, methyl 3,4,5-trimethoxybenzoate, 3,5-dimethoxy-4-hydroxybenzyl alcohol, 3,4,5-trimethoxybenzoic acid, and syringic acid. Significant composition similarity is noticed with the ultrasonic solvent extracts from *S. montana* L. honey samples [13], with predominant benzene derivatives also leading by MSYR (53.77% ± 14.59%), and followed by syringaldehyde (18.93% ± 18.86%) and benzoic acid (5.61% ± 3.60%). MSYR also predominated (54.7%) in the extract of *S. hortensis* L. honey [14], followed by benzoic acid (2.4%). Syringic acid was present with lower abundance (0.72%; 1.15%), but it was previously found in the plant [12].

Higher aliphatic alcohols were another group of abundant compounds in the extracts dominated by (*Z*)-octadec-9-en-1-ol (14.27%; 10.82%), octadecan-1-ol (5.92%; 4.42%), and hexadecan-1-ol (5.86%; 3.28%), followed by hexadecanoic acid (3.94%; 3.69%). These compounds are not important for the honey characterisation since they probably derive from the comb environment [30]. There were no similarities with the plant volatiles (mainly mono- and sesquiterpenes) investigated previously [9].

### 2.3. Color, Total Phenolic Content and Antioxidant Properties

Determined CIE Lab color coordinates, total phenolics, and antioxidant properties of *S. subspicata* Vis. honey samples determined by U*V*/*V*IS spectroscopy are presented in Table 3. The color is one of the important attribute for honey classification and the evaluation of its quality and authenticity. According to determined lightness value (L*), *S. subspicata* Vis. honey can be classified as a light honey with a medium-high value of L* (71.8). Very similar L* values were found for *Eucalyptus* and *Salix* spp. honeys [5,31], but with higher values of a*, b*, and C_ab_*. The honeys with the highest b* values were found to be sweet chestnut, strawberry-tree, savory, mint, and buckwheat [31]. The color of *S. subspicata* Vis. honey is different from *S. hortensis* L., previously investigated and described as a dark honey (L* < 55) [14].

The total phenolic content determined in *S. subspicata* Vis. honey was 425.38 mg GAE/kg, while average values for FRAP and DPPH assays were 4.26 mmol Fe^2+^/kg and 0.8 mmol TEAC/kg, respectively. Similar values were found for *S. montana* L. honey (382.3 ± 63.7 mg GAE/kg, 3.5 ± 1.0 mmol Fe^2+^/kg and 0.7 ± 0.2 mmol TEAC/kg). A strong correlation between the antioxidant activity of the honeys and their total phenolic content was previously found [32]. Similar phenolic content (406.23 ± 17.22 mg/kg) was found in *Echium vulgare* L. honey [33]. The antioxidant capacity of *S. subspicata* Vis. honey is several times higher than found for some bright unifloral honeys such as black locust, goldenrod, rapeseed, and lime [34]. Furthermore, *S. subspicata* Vis. honey showed similar or higher antioxidant activity than several honeys with higher phenolic content, such as *Coffea* spp., *Salix* spp., and *S. hortensis* L. honeys [4,5,14].

**Figure 1:**
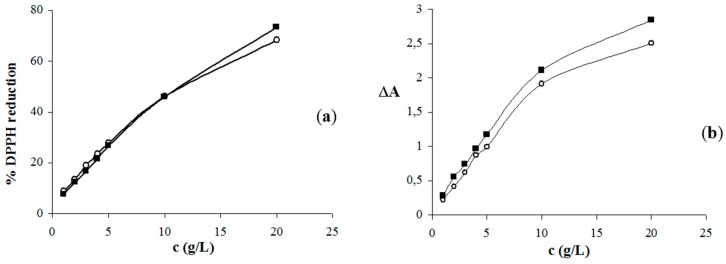
The antiradical activity (**a**) and reducing potential (**b**) of *S. subspicata* Vis. honey extracts: EX1 (○) and EX2 (■).

Literature data about the antioxidant activity of the honey volatile compounds is limited. As far as we know, besides our previous research [35,36] there are no reports on the antioxidant activity of Croatian honey ultrasonic solvent extracts. The GC-FID/MS analyses of the extracts showed that they were qualitatively very similar with small amounts, and therefore they were combined according to the type of solvent representing EX1 and EX2 (as described in Section 3.3.4.) for DPPH and FRAP assays. The radical scavenging activity of EX1 and EX2 was expressed as the percentage reduction of DPPH radical. The higher DPPH radical percentage reduction of the sample reveals higher radical scavenging (antiradical) activity. The results of EX1 and EX2 radical scavenging activity are shown in Figure 1 as the function of the extracts concentrations.

For comparison of antioxidant activity of the honey and its extracts, the average honey sample was prepared (as described in Section 3.3.4.) and tested too. The least active, at a level of only 16.5%, was the average honey sample (5% solution). Considering various modifications of the DPPH method employed by different authors, it is difficult to make direct comparisons of radical scavenging activities of *S. subspicata* Vis. honey with published data. A simple glance at Figure 1 shows that there were no significant differences among the radical scavenging ability of the extracts, although EX2 exhibited slightly better scavenging potential at the highest concentration tested. The antiradical activity of the highest concentration of EX2 (20.0 g/L) was 73.4%, in comparison with 68.3% for the same concentration of EX1. These measurements showed that both honey extracts demonstrated significantly higher antiradical activity than the honey sample at approximately 2.5 times lower concentration (the extracts concentration 20.0 g/L *vs.* the honey solution concentration 50 g/L). The radical scavenging activity is usually expressed as IC_50_, the concentration of the antioxidant that causes 50% inhibition of DPPH. IC_50_ value was approximately determined graphically from Figure 1, and for both extracts was the same 11.15 g/L. The average honey sample exhibited a FRAP value of 307.55 μM Fe(II) for 5% honey solution. Same as indicated for antiradical activity, at five times lower concentration both honey extracts demonstrated significantly higher reducing ability than the honey sample. FRAP values of 1% (10 g/L) EX1 and EX2 solutions were considerably higher: 1909.68 μM Fe(II) for EX1 and 2100.39 μM Fe(II) for EX2. Figure 1 shows the concentration-response curves for the solutions of EX1 and EX2. Similar as indicated for the antiradical activity, there were no marked differences between the extracts. The slightly more active was EX2: the reducing capacity expressed as the change in absorbance (ΔA) between the absorbance reading after 10 min and reagent blank reading for the highest tested concentration (20 g/L) was 2.846 nm, compared to 2.507 nm for the same concentration of EX1. The reasons behind the observed slight differences in antioxidant activity among the extracts probably arise from their similar chemical composition. The major phenolic compounds found in these extracts were MSYR and syringaldehyde; they were only slightly more abundant in the CH_2_Cl_2_ extract (EX2). Knowing that MSYR exhibited rather low antioxidant activity [15], somewhat higher antioxidant activity of EX2 than EX1 cannot be ascribed to MSYR, but probably to the combined activity of various, even minor, compounds present in the extracts.

## 3. Experimental Section

Ten samples of *Satureja subspicata* Vis. honey were obtained from professional bee-keepers in Croatia (the bee specie was *Apis mellifera carnica*). The honey type confirmation was carried out according to the Harmonized methods of melissopalynology [37].

### 3.1. HPLC–DAD Analysis

The honey samples were diluted in ultrapure water 1:10 (*w*/*v*), vortexed, and filtered through an RC membrane syringe filter (0.45 μm, Ø 15 mm, Phenex, Torrance, CA, USA) prior to the analysis. The analyses were performed using a HPLC-DAD Varian system ProStar fitted with a pump module 230, an autosampler module 410, and ThermoSeparation diode array detector SpectroSystem UV 6000 lp (ThermoSeparation, San Jose, CA, USA) set at 210 and 280 nm. The gradient elution was performed on Phenomenex Gemini C18 110 Å column (150 mm × 4.60 mm, 3 µm, Chemtek Analitica, Anzola Emilia, Bologna, Italy) using 0.2 M phosphoric acid (solvent A) and acetonitrile (solvent B) as mobile phase. The gradient (*v*/*v*) was formed as follows: 100% of solvent A for 5 min, then decreasing to 20% in 35 min, and finally increasing to 100% of solvent B until 60 min. The injection volume was 10 µL. The obtained chromatograms and spectra were elaborated with a ChromQuest V. 4.0 data system (ThermoQuest, Rodano, Milan, Italy). The standard solutions were prepared in methanol (kojic acid, benzoic acid, 4-hydroxyphenylacetic acid, and methyl syringate) or 0.1 M HCl/MeOH 50:50 (*v*/*v*) (tyrosine and phenylalanine), and working standard solutions were prepared in ultrapure water. The calibration curves were plotted according to the external standard method, correlating the analyte peak areas with the corresponding concentrations.

### 3.2. Ultrasonic Solvent Extraction and GC-FID/MS Analysis of the Extracts

Ultrasonic solvent extraction (USE) of the honey samples was performed in an ultrasonic bath (Clean 01, MRC Scientific Instruments, London, UK) by indirect sonication at the frequency of 35 kHz and at 25 ± 3 °C, as described in detail previously [38]. The mixture pentane/Et_2_O 1:2 (*v*/*v*) and CH_2_Cl_2_ were separately used as the solvents for the honey extraction (40 g). The obtained extracts were concentrated up to 0.2 mL by careful distillation with a Danish-Kuderna apparatus, and 1 μL of each was used for GC-FID/MS analyses.

GC-FID analyses were carried out on an Agilent Technologies (Palo Alto, CA, USA) gas chromatograph model 7890A with flame ionization detector. The chromatographic separations were performed on 30 m capillary column HP-5MS (5%-phenyl-methylpolysiloxane, Agilent J & W GC column) with 0.25 mm i.d. and 0.25 μm coating thickness. The GC conditions were: 1 μL of injected volume with split ratio 1:50; oven programmed at 70 °C for 2 min, then increased at the rate of 3 °C/min to 200 °C and held isothermal for 15 min; injector temp. 250 °C; detector temp. 300 °C; He carrier gas velocity 1 mL/min. The GC-MS analyses were performed with an Agilent gas chromatograph model 7890A fitted with mass-selective detector model 5975C (Agilent Technologies). The mass detector conditions were set up as follows: the ion source temperature 230 °C; quadrupole temperature 150 °C; transfer line temperature 270 °C; acquisition mode electron impact (EI 70 eV) by three scans·s^−1^ and mass range *m*/*z* 29–350. The separation of the compounds was performed in the same manner as for GC-FID. The individual peaks were identified by comparison of their retention indices (relative to C_9_-C_25_ *n*-alkanes) with those of available authentic samples and literature data [39], and by comparing their mass spectra with Wiley 275 MS library (Wiley, New York, NY, USA) and NIST08 (d-Gaithersburg) database. The percentage composition was calculated from the GC peak areas using the normalization method (without correction factors). All the analyses were performed in duplicate.

### 3.3. UV/VIS Analyses

All analyses (Section 3.3.1., Section 3.3.2., Section 3.3.3. and Section 3.3.4.) were performed with a UV/VIS spectrophotometer Varian series Cary 50 Scan (Varian, Leinì, TO, Italy).

#### 3.3.1. CIE L*a*b*C*_ab_h°_ab_ Chromatic Coordinates Determination

The honey samples, homogenized and transparent, were analysed in duplicate in 10 mm optical polystyrene cuvettes without any dilution (Kartell 01937). The obtained data were processed using Cary Win UV Colour Application V. 2.00 software [31]. Transmittances in a wave-length interval among 380 and 780 nm were measured using D65 illuminant with a 10° observation angle.

#### 3.3.2. Determination of Total Phenolic Content (Folin-Ciocalteu Assay)

Total phenolic content was determined spectrophotometrically by modified Folin-Ciocalteu method [15]. In each volumetric flask containing 500 µL of Folin-Ciocalteu phenol reagent, 100 µL of honey/water solution 1:5 (*w*/*v*) was added. After 5 min, 3 mL of 10% Na_2_CO_3_ (*w*/*v*) was added and the mixture was shaken and diluted with H_2_O to the volume of 10 mL. After 90 min of incubation at room temperature, the absorbance was read at 725 nm in 10 mm polystyrene cuvette (Kartell 01937) against a blank. The total phenolics content was expressed as mg/kg of gallic acid equivalent (GAE) using the calibration curve made of freshly prepared gallic acid standard solutions (10–200 mg/L).

#### 3.3.3. Honey: Total Antioxidant (FRAP Assay) and Antiradical Activity (DPPH Assay)

The reduction of ferric 2,4,6-tris(2-pyridyl)-1,3,5-triazine [Fe(III)-TPTZ] was monitored by spectrophotometric measurements of absorbance at 593 nm [15]. Twenty microlitres of the honey/water solution (1:5 *w*/*v*) was added in 10 mm polystyrene cuvettes (Kartell 01937) containing 2 mL of freshly prepared reagent (0.3123 g TPTZ, 0.5406 g FeCl_3_·6H_2_O in 100 mL acetate buffer pH 3.6). Quantitative analysis was done using the external standard method (FeSO_4_, 0.1–2 mmol/L), correlating the absorbance (λ = 593 nm) with the concentration. The results were expressed as mmol/kg of Fe^2+^.

Fifty microlitres of the honey sample diluted with ultrapure H_2_O (1:5 *w*/*v*) was added in 10 mm cuvettes (Kartell 01937) with 2 mL of DPPH solution (0.04 mmol/L in MeOH) and mixed. The spectrophotometric readings were carried out at 517 nm after 60 min incubation in the dark. The calibration curve in the range of 0.02–1.0 mmol/L was prepared for Trolox, and data were expressed as Trolox equivalent antioxidant capacity (TEAC, mmol/kg).

#### 3.3.4. Honey Extracts: Total Antioxidant (FRAP Assay) and Antiradical Activity (DPPH Assay)

Methanolic stock solutions of EX1 (combined ultrasonic extracts obtained with solvent pentane/Et_2_O 1:2 (*v*/*v*) and EX2 (combined ultrasonic extracts obtained with CH_2_Cl_2_) were prepared (the concentrations of stock solutions were 1.0, 2.0, 3.0, 4.0, 5.0, 10.0 and 20.0 g/L). Also, the average honey sample, prepared by mixing an equal quantity of each honey sample, was treated with distilled water (50 g/L) and sonicated until a clear solution was obtained.

The antiradical potential (DPPH assay) of EX1, EX2, and average honey sample was measured using the stable DPPH radical, according to the method of Brand-Williams, Cuvelier, and Berset [40] with some modifications made by Kulišić *et al.* [41]. Briefly, a stock solution (50 µL) of the antioxidant was placed in a cuvette containing 1 mL of 0.04 g/L methanolic solution of DPPH. The reaction mixture was mixed and left at room temperature in the dark for 60 min. The decrease in absorbance at 517 nm was measured against methanol blank. The absorbance of the DPPH radical without antioxidant (control) was also measured. All determinations were performed in duplicate. The radical scavenging activity of the samples was expressed in terms of the percentage inhibition of DPPH radical and was calculated as follows:

% inhibition = [(A_C_ − A_A_)/A_C_] × 100

where A_C_ is the control absorbance, and A_A_ is the antioxidant absorbance.

Total antioxidant activity was determined by the original method of Benzie and Strain with several modifications [42]. Working FRAP reagent was prepared by mixing 10 volumes of 0.3 M acetate buffer (pH 3.6) with one volume of 10 mM TPTZ (2,4,6-tripyridyl-s-triazine) solution in 40 mM HCl and with one volume of 20 mM ferric chloride (FeCl_3_·6H_2_O). Freshly prepared FRAP reagent was warmed to 37 °C, and the absorbance of the reagent blank was measured at 593 nm. All measurements were performed as follows: 50 µL of the sample was added to a cuvette containing 1 mL of freshly prepared FRAP reagent, and the reaction mixture was mixed and incubated at 37 °C for 10 min. The absorbance was measured spectrophotometrically at 593 nm. The change in absorbance (ΔA) between the absorbance reading after 10 min and reagent blank reading for each antioxidant (the honey solution and each concentration of EX1 and EX2) were calculated. The results are expressed graphically, as dose-response curves for the solutions of EX1 and EX2, and as FRAP value (µM Fe(II)) of EX1 and EX2 solutions (10.0 g/L or 1%). The FRAP value determination was performed according to the external standard method. Aqueous standard solutions of FeSO_4_·7H_2_O (100–1000 µM) were used for the calibration curve. All determinations were performed in duplicate.

## 4. Conclusions

Screening of unifloral *S. subspicata* Vis. honey revealed high content of MSYR identified by HPLC-DAD. GC-FID/MS analysis of the honey extracts additionally confirmed MSYR as a dominant chemical marker of *Satureja* spp. honey types. However, 3–4 times higher amounts of MSYR can be pointed out for distinguishing *S. subspicata* Vis. honey from other *Satureja* spp. honey types. Besides MSYR, other prephenate derivatives were found in lower abundance by HPLC-DAD and GC-FID/MS. Total phenolic content and antioxidant properties of the honey samples were similar to other *Satureja* spp. honey types. The honey extracts evaluated by FRAP and DPPH assays demonstrated higher activity (at lower concentrations) in comparison with the honey.

## Figures and Tables

**Table 1:** The compounds identified by targeted HPLC-DAD analysis of the native honey samples.

No.	Compound	Content (mg/kg)			
		Min. ^a^	Max. ^b^	Av. ^c^	SD. ^d^
1	Kojic acid	11.7	15.4	13.7	1.2
2	Tyrosine	17.2	59.0	35.6	14.8
3	Phenylalanine	330.3	52.6	167.8	80.8
4	4-Hydroxyphenylacetic acid	Nd ^e^	36.1	21.3	8.1
5	Benzoic acid	14.7	48.7	36.4	10.5
6	Methyl syringate	92.1	144.4	114.1	17.8

^a^ minimal value; ^b^ maximal value; ^c^ average value; ^d^ standard deviation; ^e^ not detected.

**Table 2:** The compounds identified by GC-FID/MS analysis of the honey extracts.

Compound	RI ^a^	I ^b^				II ^c^			
		Min. ^d^	Max. ^e^	Av. ^f^	SD. ^g^	Min. ^d^	Max. ^e^	Av. ^f^	SD. ^g^
1,4-Dimethylbenzene **	< 900	0.0	0.3	0.14	0.11	0.0	0.1	0.06	0.06
1,3-Dimethylbenzene **	< 900	0.0	0.2	0.08	0.08	-	-	-	-
Decane	1000	0.0	0.1	0.04	0.06	-	-	-	-
Benzoic acid	1162	0.5	4.7	2.22	2.21	0.5	4.3	1.76	1.40
2,4-Dimethylbenzaldehyde **	1178	-	-	-	-	0.0	0.1	0.04	0.05
3,7-Dimethylocta-1,5-dien-3,7-diol	1191	-	-	-	-	0.0	0.1	0.06	0.06
3,4,5-Trimethylphenol **	1331	0.0	0.1	0.06	0.06	-	-	-	-
Eugenol	1360	0.0	0.1	0.06	0.06	0.0	0.9	0.34	0.34
Tetradecane	1400	0.0	0.1	0.06	0.06	0.0	0.5	0.13	0.17
β-Caryophyllene	1425	-	-	-	-	0.0	0.1	0.04	0.05
4-Methyl-2,6-bis(1,1-dimethylethyl)-phenol	1520	0.2	0.5	0.31	0.13	0.0	0.6	0.16	0.24
Methyl 4-methoxybenzoate	1527	0.0	1.1	0.43	0.60	-	-	-	-
Hexadecane	1600	0.0	0.6	0.18	0.27	0.0	1.3	0.42	0.48
3,4,5-Trimethoxybenzaldehyde	1608	000	0.4	0.14	0.19	0.0	0.7	0.20	0.31
Syringaldehyde	1662	2.1	11.1	7.30	3.96	4.0	14.1	8.80	3.27
Methyl 3,4,5-trimethoxybenzoate *	1675	0.0	4.1	0.94	1.78	0.0	0.5	0.22	0.24
3,5-Dimethoxy-4-hydroxybenzyl alcohol *	1721	-	-	-	-	0.0	1.8	0.73	0.88
Methyl syringate	1744	32.3	56.1	46.68	10.82	41.7	60.1	52.98	7.66
3,4,5-Trimethoxybenzoic acid **	1775	0.0	1.2	0.34	0.53	0.0	2.9	0.88	1.18
Syringic acid	1840	0.0	1.4	0.72	1.02	0.0	4.1	1.15	1.63
Hexadecan-1-ol	1882	2.8	9.4	5.86	2.70	0.6	5.6	3.28	1.66
Hexadecanoic acid	1963	1.0	9.4	3.94	3.35	1.0	12.7	3.69	4.14
Eicosane	2000	-	-	-	-	0.0	1.3	0.32	0.54
Octadecanal	2024	-	-	-	-	0.0	1.0	0.35	0.43
(*Z*)-Octadec-9-en-1-ol	2073	6.7	27.7	14.27	9.33	5.9	18.6	10.82	4.41
Octadecan-1-ol	2084	2.4	10.4	5.92	2.97	2.1	6.9	4.42	1.57
Heneicosane	2100	0.0	4.0	0.12	0.18	0.0	5.5	1.40	2.11
Octadecanoic acid	2178	0.0	0.9	0.22	0.36	0.0	1.0	0.23	0.41
(*Z*)-Octadec-9-enoic acid	2181	-	-	-	-	0.0	0.9	0.22	0.36
Docosane	2200	-	-	-	-	0.0	0.7	0.23	0.29
Tricosane	2300	-	-	-	-	0.0	0.9	0.27	0.36

^a^ retention indices relative to C_9_–C_25_ *n*-alkanes, ^b^ ultrasonic extracts with the solvent pentane/Et_2_O 1:2 (*v*/*v*); ^c^ ultrasonic solvent extract with CH_2_Cl_2_; ^d^ minimal percentage; ^e^ maximal percentage; ^f^ average percentage; ^g^ standard deviation; ** correct isomer is not determined; * tentatively identified.

**Table 3:** The characteristics of *S. subspicata* Vis. honey samples.

	CIE Lab Colour Coordinates					TP ^f^ (mg GAE/kg)	FRAP ^g^ (mmol Fe^2+^/kg)	DPPH ^h^ (mmol TEAC/kg)
	L* ^a^	a* ^b^	b* ^c^	C* _ab_^d^	h°_ab_^e^			
Min. ^i^	67.0	10.7	65.7	66.6	79.0	347.14	2.93	0.57
Max. ^j^	76.2	14.5	74.8	76.2	81.2	490.04	6.01	1.18
Av. ^k^	71.8	12.5	71.6	72.6	80.1	425.38	4.26	0.80
SD. ^l^	3.1	1.4	2.9	3.0	0.8	44.20	0.90	0.19

^a^ lightness. ^b^ indicates red for positive value and green for negative value; ^c^ indicates yellow for positive value and blue for negative value; ^d^ chroma.; ^e^ hue, deg.; ^f^ Total phenols (TP) value is expressed as gallic acid equivalent (GAE); ^g^ FRAP value expressed as millimolar concentration of Fe^2+^ obtained from a dilution of FeSO_4_ having an equivalent antioxidant capacity as the honey solution; ^h^ DPPH value expressed as millimolar concentration of TEAC obtained from a dilution of Trolox having an equivalent antiradical capacity as the honey solution; ^i^ minimum value; ^j^ maximum value; ^k^ average value; ^l^ standard deviation.
